# Gregarine parasites are adapted to mosquito winter diapause

**DOI:** 10.1186/s13071-022-05365-w

**Published:** 2022-07-11

**Authors:** Edwige Martin, Laurent Vallon, Camille Da Silva Carvalho, Maxime Girard, Guillaume Minard

**Affiliations:** UMR Ecologie Microbienne, Université Claude Bernard Lyon 1, CNRS, INRAe, VetAgro Sup, 69622 Villeurbanne, France

**Keywords:** *Ascogregarina*, *Aedes albopictus*, Thermal tolerance, Apicomplexa

## Abstract

The Asian tiger mosquito *Aedes albopictus* is one of the most invasive species of mosquito. The prevalence of its apicomplexan gregarine parasite *Ascogregarina taiwanensis* is high in natural populations across both temperate and tropical regions. However, the parasite’s oocysts cannot colonize the insect host during winter, when the mosquito lays diapausing eggs. It is therefore unclear if the parasite can survive outside of its insect host during the cold season in temperate regions. Oocysts stored for 1 month at a low temperature (representative of the temperatures that occur during periods of mosquito diapause) were as infectious as fresh oocysts, but those stored for the same period of time at a higher temperature (representative of the temperatures that occur during periods of mosquito activity) were uninfectious. We therefore suggest that the parasite has evolved traits that maximize its maintenance during periods of mosquito dormancy, while traits that would enable its  long term survival during periods of mosquito activity have not been selected for.

The phylum Apicomplexa, which comprises heterotrophic animal parasites, evolved from photosynthetic marine algae [1]. The Apicomplexa have been intensively studied because important human pathogens belong to this phylum, such as *Toxoplasma* and *Plasmodium*. Gregarine parasites (Gregarinasina) are of great interest from an evolutionary perspective since their lineage is the most ancient of the Apicomplexa. They are highly diverse and infect a large number of invertebrates across marine, freshwater and terrestrial ecosystems [2]. Their interactions with their hosts are complex, and range from mutualism to parasitism.

In mosquitoes, only one gregarine genus has been reported, namely *Ascogregarina* [3]. Species of the genus *Ascogregarina* mainly colonize *Aedes* spp., but also colonize *Tripteroides dofleini* and *Armigeres subalbatus*, and show high host specificity. They are often considered weakly pathogenic to their natural hosts but harmful to other species [4]. The Asian tiger mosquito *Aedes albopictus* is one of the most invasive species of mosquitoes in the world [5]. Originating in Asia, it has efficiently colonized every continent, except for Antarctica, over the past century [6]. Its ability to lay diapausing eggs which can remain dormant during the winter has enabled it to survive low temperatures and colonize temperate regions [7]. Its gregarine parasite *Ascogregarina taiwanensis* has a remarkable prevalence in both native and introduced populations [8–11], although some exceptions have been reported [12]. The parasites are released as oocysts (a means of environmental resistance) by adult mosquitoes when these emerge, die or defecate in freshwater [3]. The oocysts infect 1st instar mosquito larvae through ingestion in their freshwater habitat, and the biological cycle of the parasite, which occurs within the digestive tract of the insect (i.e. the gut lumen, intestinal epithelium and lumen of the Malpighian tubules) is regulated by the development of the mosquito [13, 14]. It is, however, unclear whether the parasite can remain infectious during the winter period. In this study, we aimed to test the infective ability of oocysts under winter and summer conditions.

We generated a line of mosquitoes from a focal population collected in September 2017 in Villeurbanne and Pierre-Bénite (France). The mosquitoes were reared under laboratory conditions at 28 °C. The presence of *A. taiwanensis* in the line was confirmed by diagnostic polymerase chain reaction following the protocol described by Reyes-Villanueva et al. [15], and by microscopic observation of the gut in pupae and Malpighian tubules in adults. The parasite was maintained in the line over 22 generations by (i) sprinkling the crushed bodies of infected adults over the water in the containers holding the larvae, (ii) not storing eggs, and (iii) never changing the water in the containers before the 2nd instar stage. A non-infected line was generated from the same focal population of mosquitoes and maintained for 18 generations. This was achieved by (i) treating F_1_ mosquito eggs with griseofulvin (1 mg ml^−1^), a fungicide that shows antiparasitic activity against gregarines [16]; and (ii) changing the water in which the 1st instar larvae were maintained. We verified the absence of the parasite by microscopy.

To test our hypothesis, we exposed 100 uninfected mosquito larvae to fresh oocysts or oocysts stored for 1 month at a low temperature (4 °C), or oocysts stored for 1 month at a higher temperature (26 °C). The oocysts were obtained by crushing 100 infected adult mosquitoes in 45-ml sterile water in a Dounce tissue grinder tube (mean ± SD = 2.56 ± 2.14 × 10^6^ oocysts). The contents of each tube were then separated into three 15-ml tubes. The contents of one of these tubes was used to immediately re-infect mosquitoes, while the contents of the other tubes were stored at 4 °C or 26 °C. The experiment was replicated for three different mosquito batches per condition. A negative control consisting of uninfected mosquitoes was prepared to ensure that there was no oocyst material in the test population. We then counted the number of oocysts that had colonized the adult individuals that emerged under each of the test conditions. Survival to immature stages was slightly higher for uninfected individuals (mean ± SD = 87.67 ± 3.29%), individuals colonized with parasites stored at 26 °C (mean ± SD = 93.67 ± 0.58%) and individuals immediately reinfected with parasites (mean ± SD = 85.67 ± 5.03%) compared to those reinfected with parasites stored at 4 °C (mean ± SD = 75.33 ± 3.06%). 

Individual mosquitoes were crushed with a sterile pestle in 100 µl of sterile water (Gibco, France). Oocysts were counted in 10 haemocytometer cells per mosquito at ×400 magnification under a microscope (Leica, Germany). Number of oocysts per mosquito was calculated using the following equation: $n({\text{oocysts/mosquito}})={\text{Water volume}} \times {{\frac{\text{Average number of oocyst per cell}}{{\text{Cell volume}}}}}$. Oocyst counts were determined for a total of 10 males and 10 females per condition and per replicate (i.e. a total of 240 individuals). 

Statistical analyses were performed with R software v. 4.0.2 (R Development Core Team). The data were plotted using the ggplot2 and ggpubr R packages. 

Interestingly, no oocysts were retrieved from the majority of mosquitoes infected with oocysts stored at 26 °C (Fig. 1), or from the uninfected individuals. These results differed from those for mosquitoes infected with fresh oocysts or oocysts stored at 4 °C. These observations were consistent across sexes and replicate batches. Since the data were zero-inflated, the oocyst count data were transformed into presence–absence for the analysis, where presence indicated that an individual was parasitized and absence indicated that it was not. Variation in the oocyst infection rate between replicate batches, male and female mosquitoes, and experimental conditions were modelled with a generalized linear model with a binomial distribution using the brglm2 R package. The influence of each explanatory variable on the response variables was tested by type II Wald χ^2^ with the car R package. This showed that oocyst infection rate was significantly influenced by the experimental condition (Wald χ^2^, *P* < 2 × 10^–16^) and by the sex (Wald χ^2^, *P* = 0.02) of the mosquito in interaction with the replicate batch (Wald χ^2^, *P* = 0.02; Table 1). Therefore, independent analyses were repeated for each sex separately. Oocyst infection rate variation among mosquitoes of both sexes was significantly affected by the experimental conditions tested (Wald χ^2^, females, *P* < 2 × 10^–16^; males, *P* = 7.98 × 10^–15^), and differences were also observed between batches for males (Wald χ^2^, *P* = 3.78 × 10^–4^; Table 1). After averaging the values of the batch replicates, post hoc comparisons of pairwise experimental conditions were performed using Tukey honest significant difference (HSD) test with the emmeans R package. These pairwise comparisons indicated that females and males showed consistent patterns despite the batch effect observed for the males (Table 2). The pairwise comparisons showed that oocytes stored in water under a low temperature were still infectious, and that infection rates under this condition did not significantly differ from those in which fresh oocysts were used to infect mosquitoes (Tukey HSD, females, *P* = 0.3; males, *P* = 0.98). In contrast, rates of infection with oocysts stored in 26 °C water were consistently lower than those with fresh oocysts or oocysts stored at 4 °C (Tukey HSD, *P* < 10^–2^ for each comparison across both sexes), and did not significantly differ from those of uninfected control individuals (Tukey HSD, males,* P* = 0.99; females, *P* = 0.99).

**Fig. 1:**
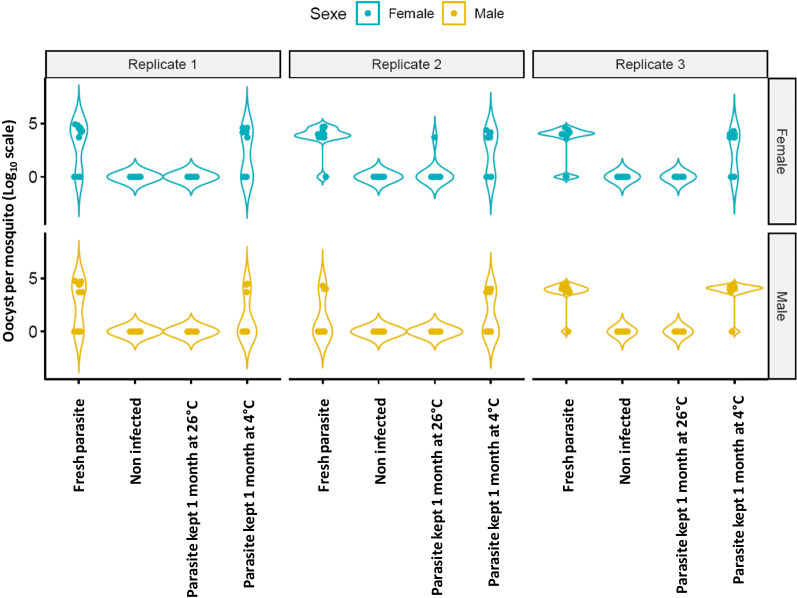
Number of oocysts per adult mosquito across the experimental conditions. Females (blue) and males (yellow) were tested across three independent replicate batches. The violin plots show the log_10_ of total oocyst number + 1 across the experimental conditions. In the Fresh oocysts treatment, infected mosquitoes were crushed into sterile water which was immediately used to seed the water in the tray containing the unparasitized 1st instar larvae. Non-infected mosquitoes were unparasitized and used as a control to confirm that no residual parasites had colonized the test population. In the Parasite kept 1 month at 26 °C or 4 °C treatments, infected mosquitoes were crushed in sterile water and stored for 1 month at 26 °C or at 4 °C, respectively before being seeded to tray containing unparasitized 1st instar larvae. Larvae were reared to adult mosquitoes before parasitism measurement

**Table 1:** Factors explaining variation in the oocyst infection rate

Dataset	Response variable	Explanatory variable	Wald χ^2^	*df*	*P*-value	Significance
Full	Oocyst infection (presence/absence)	Condition	142.668	3	< 2 × 10^–16^	***
		Batch	7.719	2	0.02	*
		Sex	2.438	1	0.12	
		Condition × Batch	1.960	6	0.92	
		Condition × Sex	1.254	3	0.74	
		Batch × Sex	8.110	2	0.02	*
		Condition × Batch × Sex	− 9.087	6	1	
Females	Oocyst infection (presence/absence)	Condition	76.508	3	< 2 × 10^–16^	***
		Batch	1.849	2	0.4	
		Condition × Batch	− 2.976	6	1	
Males	Oocyst infection (presence/absence)	Condition	68.730	3	7.98 × 10^–15^	***
		Batch	15.764	2	3.78 × 10^–4^	***
		Condition × Batch	− 4.150	6	1	

* *P* < 0.05, ** *P*  < 0.01, *** *P* < 0.001

**Table 2:** Pairwise comparisons of oocyst infection rate between conditions

Dataset	Condition 1 in the comparison	Condition 2 in the comparison	*z*-ratio	*P*-value	Significance
Females	Parasite stored for 1 month at 26 °C	Parasite stored for 1 month at 4 °C	− 3.353	4.4 × 10^–3^	**
	Parasite stored for 1 month at 26 °C	Infection with fresh oocysts	− 4.344	1 × 10^–4^	***
	Parasite stored for 1 month at 26 °C	Non-infected mosquitoes	0.341	0.99	
	Parasite stored for 1 month at 4 °C	Infection with fresh oocysts	− 1.757	0.30	
	Parasite stored for 1 month at 4 °C	Non-infected mosquitoes	3.459	3 × 10^–3^	**
	Infection with fresh oocysts	Non-infected mosquitoes	4.372	1 × 10^–4^	***
Males	Parasite stored for 1 month at 26 °C	Parasite stored for 1 month at 4 °C	− 3.351	4.5 × 10^–3^	**
	Parasite stored for 1 month at 26 °C	Infection with fresh oocysts	− 3.601	1.8 × 10^–3^	**
	Parasite stored for 1 month at 26 °C	Non-infected mosquitoes	0	1	
	Parasite stored for 1 month at 4 °C	Infection with fresh oocysts	− 0.396	0.98	
	Parasite stored for 1 month at 4 °C	Non-infected mosquitoes	3.351	4.5 × 10^–3^	**
	Infection with fresh oocysts	Non-infected mosquitoes	3.601	1.8 × 10^–3^	**

* *P* < 0.05, ** *P*  < 0.01, *** *P* < 0.001

In temperate regions, the active period of mosquitoes ranges from April/May to October/November, while during the rest of the year mosquitoes lay diapausing eggs that remain dormant until the following active period [17]. *Ascogregarina taiwanensis* colonizes every developmental stage of mosquitoes except for the egg stage [13]. Therefore, it cannot infect its mosquito hosts during the period of dormancy, and thus persists as a free-living oocyst until mosquito eggs hatch. However, one can hypothesize that the parasite may infect its host during the inactive period if life stages other than eggs are maintained during the winter. Adult hibernation has been reported in some mosquito species [18] but not, to the best of our knowledge, in *Ae. albopictus*. Mosquitoes can pursue their cycle over the winter if they find warm refuges. However, *Ae. albopictus* mostly colonizes open habitats [19]. The overwintering of mosquitoes in greenhouses have been reported in the Netherlands, but this remains a solitary example of this behaviour [20]. Concrete tanks were used as winter refuges for larvae and pupae in northern Vietnam [21], but the winter temperatures in this subtropical region average 14 °C, which is sufficiently high to allow the slow but complete development of the mosquito. In contrast, open bodies of water in temperate regions can reach lower temperatures in winter. 

The prevalence of *Ascogregarina taiwanensis* in* Aedes albopictus* can reach 100% in temperate regions [11]. This finding is inconsistent with low survival of the parasite in mosquito refuges during the winter. However, based on our results, we can propose an explanation for this discrepancy. Since the oocysts showed no decrease in infectivity when they were stored in water at a low temperature (at least 1 month at 4 °C), we suggest that the parasite is adapted to survive outside of its host during the latter’s period of inactivity. It is interesting to note that the survival of the parasite at the higher temperature tested, which is representative of temperatures that occur during the period of mosquito activity, was poor. A possible explanation for this is that the parasite is more likely to encounter a mosquito host in warmer periods, and thus there may be less selective pressure for resistance in oocysts at temperatures prevalent during the mosquito’s active season.

 In conclusion, our study provides an example of parallel phenology which may explain the success of a neglected, but widespread, invasive parasitic species in infecting its mosquito host. This profile resembles that of *Cryptosporidium*, an apicomplexan parasite of mammals that is transmitted in water. In that model, infectivity remained stable for at least 12 weeks when the oocysts were stored in water at 4 °C, but dropped dramatically after 6 weeks when the oocysts were stored at 25 °C [22]. This type of pattern could have epidemiological consequences, since transmission rates of viruses and filarial nematodes in *Aedes* mosquitoes have been shown to vary according to the degree to which the mosquitoes have been parasitized by *Ascogregarina* spp. [23, 24]*.*

## Data Availability

The datasets supporting the conclusions of this article are included within the article and in its additional file.
